# Clinical Value of Serum Neuron-Specific Enolase Combined with Serum S100B Protein in the Diagnosis of Systemic Lupus Erythematosus

**DOI:** 10.1155/2022/9390991

**Published:** 2022-05-09

**Authors:** Jing Chen, Yan cheng, Xiaogang Zhang, Fukun Wang, Xia Chuai

**Affiliations:** ^1^Department of Clinical Laboratory, The 980th Hospital of PLA Joint Logistics Support Force, Shijiazhuang 050082, China; ^2^Department of Basic Medical Laboratory, The 980th Hospital of PLA Joint Logistics Support Force, Shijiazhuang 050082, China; ^3^Department of Rheumatology and Immunology, The 980th Hospital of PLA Joint Logistics Support Force, Shijiazhuang 050082, China; ^4^Department of Pathogenic Biology, Hebei Medical University, Shijiazhuang 050082, China

## Abstract

**Objective:**

To investigate the clinical value of serum neuron-specific enolase (NSE) combined with serum S100B protein in the diagnosis of systemic lupus erythematosus (SLE).

**Methods:**

Sixty patients with SLE treated in our hospital from January 2019 to April 2021 were enrolled as the study group. According to the degree of activity, the study group was assigned into three groups: mild activity group (*n* = 20), moderate activity group (*n* = 20), and severe activity group (*n* = 20). A total of 60 healthy people who underwent physical examination in our hospital in the same period were enrolled as the control group. The NSE and serum S100B protein were detected in the two groups, and the correlation between serum nerve-specific enolase and serum S100B protein and the clinical value in the diagnosis of SLE were analyzed.

**Results:**

First of all, we compared the general data of the two groups. There was no significant difference in sex, age, marital status, and education level, and no significant difference was exhibited (*p* > 0.05). There was no significant difference in sex, age, marital status, and education level among mild activity group, moderate activity group, and severe activity group, and no significant difference in data was exhibited (*p* > 0.05). Secondly, we compared the levels of serum S100B protein and NSE. The levels of serum S100B protein and NSE in the study group were higher compared to the control group (*p* < 0.05). The levels of serum S100B protein and NSE in patients with different activity levels of SLE were compared. The levels of serum S100B protein and NSE in mild activity group < moderate activity group < severe activity group were significantly different (*p* < 0.05). Correlation analysis between serum S100B, NSE levels, and SLE activity indicated that serum S100B and NSE levels were positively correlated with SLE activity. With the increase of SLE activity, serum S100B and NSE levels gradually increased, and the data difference was statistically significant (*r* = 0.855, 0.844, *p* < 0.05). Finally, we established the logistic prediction model, take the probability of generating prediction as the analysis index, and draw the ROC curve to evaluate the diagnostic value of different combinations to SLE. The highest AUC and sensitivity of the two indexes in the diagnosis of SLE were 0.773 and 0.836, respectively. The levels of serum S100B protein and NSE have a certain value in the diagnosis of SLE, while the combined diagnosis is of higher value, sensitivity, and specificity in the diagnosis of SLE.

**Conclusion:**

Serum S100B protein and NSE are very sensitive indexes to judge the damage of central nervous system. However, due to the small number of cases in this study, there were as many as 19 kinds of NPSLE classification, so the relationship between serum S100B protein, NSE levels, and various NPSLE and their exact application value in diagnosing the disease and judging the prognosis needs to be confirmed by expanding the number of cases.

## 1. Introduction

Systemic lupus erythematosus (SLE) is a diffuse connective tissue disease mediated by autoimmunity, which is characterized by the loss of self-tolerance, the formation of specific antibodies against autoantigens, and the deposition of immune complexes formed by antigen-antibody binding, leading to type III allergy, which mediates tissue and organ damage [1]. This brings about great heterogeneity in the clinical manifestations of patients with SLE, which can involve any tissue and organ of the body, and indicates chronic recurrence or remission alternation [2]. In recent years, with the deeper understanding of SLE, the damage state of SLE to human is becoming clear. Rash is the earliest symptom, and it is easy to invade the kidney, lung, and central nervous system (CNS) in the later stage. Studies have indicated that SLE not only causes serious extensive damage, but even is life-threatening [3]. The cost of SLE treatment in the later stage of the disease is very high, and it will cause patients to lose their ability to work, resulting in greater indirect economic losses. Meanwhile, the life quality of SLE patients is greatly reduced, the population burden is increased, and the survival rate is low, ranging from 95% in 5 years to 78% in 20 years [4]. The age of onset of SLE is mainly between 25 and 45 years old, and the ratio of male-to-female incidence is about 1 : 9 [5]. It is reported that the global incidence of SLE is about 4.5/100,000, and the prevalence rate is about 32/100,000, which brings a great burden to the economic development of the country [6]. At present, the treatment of SLE mainly emphasizes the use of individualized treatment, including nonsteroidal anti-inflammatory drugs, hydroxychloroquine, corticosteroids, antiproliferative drugs, cyclophosphamide, and biological agents [6]. A single clinical manifestation is difficult to guide clinical drug administration, and it is necessary to continuously monitor the changes of patients' condition to provide reference for treatment. Therefore, accurate evaluation of SLE activity is of great significance for the treatment, and an accurate index is needed to judge the disease activity of patients. SLEDAI-2000 is mostly employed to evaluate the disease activity of patients, and there are also some evaluation indexes, such as BILAG and SLAM [7]. This kind of index can accurately judge the change of SLE, but there are still some deficiencies, such as untimely, unstable, and affected by human factors. SLE has the characteristics of high harm, high burden, difficult to cure, and repeated delay, which makes scholars pay close attention to exploring a rapid, cheap, accurate, and efficient biochemical index to reflect the changes of patients' condition, which has become one of the urgent problems to be solved [8].

The markers commonly employed in SLE-assisted diagnosis include serum NSE and S100B protein [9]. NSE is secreted by neurons, peripheral neuroendocrine tissues, and APUD cells. Studies have indicated that elevated NSE is found in patients with SLE, and the incidence of elevated concentration is higher in advanced diseases [10]. Other studies have found that serum NSE levels are associated with poor histological types [11]. The specificity of serum NSE in the diagnosis of SLE is lower compared to sensitivity (also increases in many other tumors including lymphoma), while the specificity of S100B protein is higher compared to sensitivity. Therefore, the diagnostic criteria of SLE should be combined with S100B protein. However, the sensitivity and specificity of these two markers in SLE are rarely studied. Meanwhile, previous studies have reported that the level of preconditioning NSE is related to the prognosis of SLE, but the correlation between the dynamic changes of NSE during diagnosis and prognosis is not clear [11]. S100B protein is mainly derived from astrocytes, oligodendrocytes, and Schwann cells in the CNS, accounting for about 96% of the total [12]. And S100B protein is a small calcium-binding protein, which is activated instantly when it binds to calcium ion, which can induce conformational changes in the S100B C-terminal, causing proteins to be exposed to hydrophobic plaques. Their activities are regulated by interacting with a series of target proteins, such as enzymes, enzyme substrates, cytoskeletal proteins, connectors/scaffolds, transcription factors, ion channels, and ubiquitin E3 ligases. Therefore, S100B is involved in the regulation of energy metabolism, transcription, protein phosphorylation, cell proliferation, survival, differentiation, and calcium homeostasis [13]. In general, the level of S100B protein in cerebrospinal fluid and blood of healthy people is very low; when there is brain injury, the level of S100B protein in cerebrospinal fluid and blood increases. Studies have indicated that the concentration of S100B protein in serum can be employed as a sensitive and specific marker in the evaluation of SLE [14]. In this study, we focus on the clinical value of serum nerve-specific enolase combined with serum S100B protein in the diagnosis of SLE.

## 2. Patients and Methods

### 2.1. General Information

Sixty patients with SLE treated in our hospital from January 2019 to April 2021 were enrolled as the study group. According to the degree of activity, the study group was assigned into three groups: mild activity group (*n* = 20), moderate activity group (*n* = 20), and severe activity group (*n* = 20). A total of 60 healthy people who underwent physical examination in our hospital in the same period were enrolled as the control group. In the control group, the age was 21–63 years old with an average age of 35.64 ± 3.31 years, including 33 males and 27 females, while in the study group, the age was 23–66 years old, with an average age of 35.12 ± 3.53 years, including 31 males and 29 females. There was no statistical significance in the general data of the two groups. This study was permitted by the Medical Ethics Association of our hospital, and all patients signed informed consent.

#### 2.1.1. Diagnostic Criteria

SLE classification criteria revised by American Society of Rheumatology (ACR) in 1997 were employed for diagnosis [15]. The classification standard contains a total of 11 criteria. During the observation period, as long as there were 4 or more of the diagnostic criteria (meanwhile or successively), the patient could be confirmed as SLE. SLE disease activity score was evaluated by SLE activity index 2000 (SLEDAI2000). The score was based on the presence of 24 characteristics in 9 organ systems in the past 10 days. The judgment of SLEDAI-2000 scores on the condition of SLE was as follows: 0–4: basically inactive; 5–9: mild activity; 10–14: moderate activity; and ≥ 15: severe activity.

#### 2.1.2. Inclusion Criteria

(1) Age ≥18 years old; (2) all included patients were diagnosed according to relevant criteria; and (3) all patients should be diagnosed for the first time.

#### 2.1.3. Exclusion Criteria

(1) Patients with other autoimmune diseases; (2) patients with malignant tumors in organs and tissues; (3) patients with liver diseases, such as viral hepatitis and liver cirrhosis; and (4) patients with anemia.

### 2.2. Treatment Methods

In this study, we collected case-related information, including sex, age, marital status, and education, and compared the levels of serum NSE and S100B protein in patients with SLE.

### 2.3. Observation Index

#### 2.3.1. Detection of Serum NSE Level

The level of serum NSE was detected 1 day before operation, 1 day, 3 days, and 7 days after operation, the peripheral venous blood 5 mL was collected, and the serum was separated by centrifugation 10 min with a radius of 5 cm and 3000 rpm and stored in a cryogenic refrigerator (−40°C, Thermo Company, USA). The level of serum NSE was measured by Electrochemiluminescence, and Electrochemiluminescence kit E-602 was purchased from Roche Company.

#### 2.3.2. Detection of Serum S100B Protein

Serum S100B protein was detected by Electrochemiluminescence provided by New Industrial Co., Ltd. (China), according to the instructions.

### 2.4. Statistical Analysis

The sample size was estimated by PASS15 software. The data input was exported by hospital HIS system, the data was cleaned by SAS University Edition, and the data was analyzed by SPSS18.0. Counting data are expressed in terms of rate, and *χ*^2^ test was employed for comparison between groups; mean ± standard deviation ($\bar{x}$ ±*s*) was employed for measurement data of normal distribution, *t*-test or one-way analysis of variance (ANOVA) was employed for comparison between groups, and SNK-*q* test was employed for pairwise comparison among three groups. The measurement data that did not conform to the normal distribution were represented by median (*M*) and quartile spacing (IQR), that was, *M* (P25∼P75). Mann–Whitney *U* rank sum test was employed for intergroup comparison, and Kruskal–Wallis *H* test was employed for multigroup comparison. The correlation between variables was analyzed by Spearman's correlation. To evaluate the diagnostic value of a single laboratory index, draw the subject working characteristics (ROC), find the best segmentation point, and calculate the corresponding sensitivity, specificity, and accuracy to evaluate the diagnostic value of multi-index joint detection, it was necessary to establish the logistic prediction model, generate the prediction probability as the analysis index, draw the ROC curve, and determine the corresponding sensitivity, specificity, and accuracy. The test level *α* = 0.05 (*p* < 0.05) indicated that the difference was statistically significant.

## 3. Results

### 3.1. Comparison of General Data

First of all, we compared the general data of the two groups. There was no significant difference in sex, age, marital status, and education level, and no significant difference was exhibited (*p* > 0.05). There was no significant difference in sex, age, marital status, and education level among mild activity group, moderate activity group, and severe activity group, and no significant difference in data was exhibited (*p* > 0.05). All the data results are provided in Table 1.

### 3.2. Comparison of Serum S100B Protein and NSE Levels

Secondly, we compared the levels of serum S100B protein and NSE. The levels of serum S100B protein and NSE in the study group were significantly higher compared to the control group (*p* < 0.05). All the data results are provided in Table 2.

### 3.3. Serum S100B Protein and NSE Levels in Patients with SLE with Different Activity

Thirdly, we compared the levels of serum S100B protein and NSE in patients with different activity levels of SLE. The levels of serum S100B protein and NSE in mild activity group < moderate activity group < severe activity group were significantly different (*p* < 0.05). All the data results are provided in Table 3.

### 3.4. Correlation between Serum S100B Protein, NSE Levels, and SLE Activity

**Figure 1:**
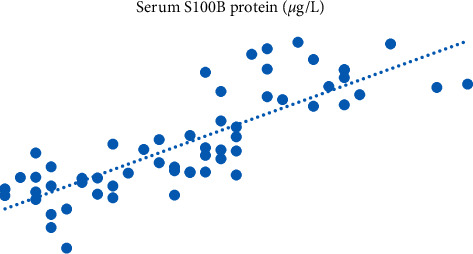
Pearson's correlation analysis between serum S100B and SLE activity.

**Figure 2:**
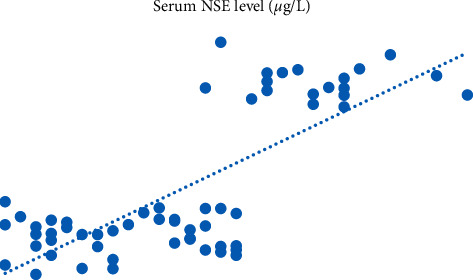
Pearson's correlation analysis between serum NSE and SLE activity.

Then, we carried out Pearson's correlation analysis between serum S100B, NSE levels, and SLE activity. Pearson's analysis indicated that serum S100B and NSE levels were positively correlated with SLE activity. With the increase of SLE activity, serum S10B and NSE levels gradually increased; the difference exhibited was statistically significant (*p* < 0.05). All the data results are shown in Figures 1 and 2.

### 3.5. ROC Curve of Serum S100B Protein and NSE Level in Diagnosis of SLE

**Figure 3:**
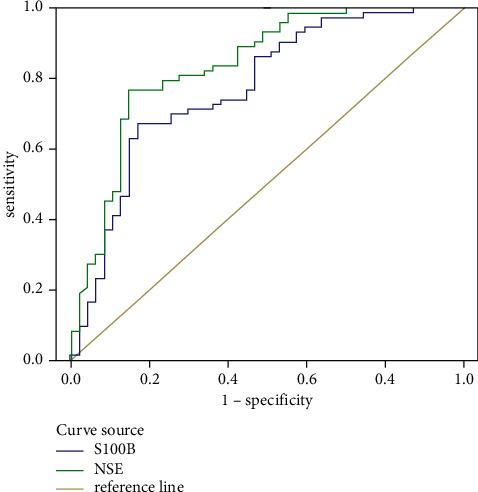
ROC curve of serum S100B protein and NSE levels in the diagnosis of SLE disease.

Finally, we established the logistic prediction model, took the probability of generating prediction as the analysis index, and draw the ROC curve to evaluate the diagnostic value of different combinations to SLE. The highest AUC and sensitivity of the two indexes in the diagnosis of SLE were 0.773 and 0.836, respectively. The levels of serum S100B protein and NSE had a certain value in the diagnosis of SLE, while the combined diagnosis was of higher value, sensitivity, and specificity in the diagnosis of SLE (*p* < 0.05). All the data results are shown in Figure 3.

## 4. Discussion

SLE is a classic systemic autoimmune disease with complex clinical manifestations, which can invade the connective tissues of the whole body and damage multiple systems and organs [15]. SLE occurs all over the world and the patients are mainly young women with a male-to-female ratio as high as 1 : 9 [16]. According to the latest data of the Chinese SLE Research Cooperation Group, the global average prevalence rate of SLE is 12–39/100000 and the prevalence rate of Chinese population is 30–70/100000, second only to blacks (100/100000), ranking second in the world [17]. The pathogenesis of SLE is not completely clear. At present, it is believed that is related to heredity, environment, immunity, and hormones [18]. SLE as a variety of diseases with abnormal changes in the autoimmune system, immune deficiency or abnormal regulation, and other autoimmune characteristics has been one of the hotspots of etiological research [17,18]. Recent studies have found that helper T cells 17 (Th17), follicular helper T cells, and regulatory B cells are closely related to the pathogenesis of SLE [19]. Th17 is a kind of T helper cells that can secrete proinflammatory factor IL-17. The expression of Th17 and IL-17 is increased in serum of patients with SLE, especially in patients with renal involvement. It is found that activation of Sy *k* and NF-*κ*B pathway could increase the secretion of IL-17A and IL-17F and further induce the occurrence of SLE. Meanwhile, Tasneem et al. found that inducible costimulator (ICOS) expressed by Th cells and its secreted cytokines IL-21 and IL-10 could assist B cells to produce autoantibodies [20]. Rodrigo Poubel Vieira de found that Breg was abnormally increased in patients with SLE, and the secretion of IL-10 was involved in the regulation of immune response [21]. Although the pathogenesis of SLE has been not clear, SLE is essentially immune-mediated inflammatory response, and chronic inflammatory response is an important clinical feature of SLE [17]. In 2009, SLE International Clinical Cooperation Group (SLLCC) modified the new standard to meet the clinical and research needs and supplement and modify the deficiencies in the ACR1997 annual standard. Through the verification and comparison of SLLCC diagnostic criteria and 1997 ACE diagnostic criteria, it is known that the sensitivity and specificity of SLLCC diagnostic criteria are 98% and 91%, while those of ACR diagnostic criteria are 88% and 98%, respectively. The former has higher sensitivity and can effectively reduce the missed diagnosis rate of SLE [22]. The diagnostic criteria of SLLCC have reflected the progress of understanding of SLE in recent decades, but SLE does not always cause multiple systemic damage; sometimes, it only indicates chronic cutaneous lupus erythematosus, while only 5% of chronic cutaneous lupus erythematosus develops into systemic damage, so SLLCC diagnostic criteria are not consistent with all SLE types of diagnosis. Therefore, looking for more specific standards is the development direction of this SLE research field in the future.

NSE exists specifically in neurons and neuroendocrine cells [22]. It is a key enzyme involved in intracellular glycolysis and is usually not secreted. It is considered to be an important factor in the pathophysiological process of CNS diseases and autoimmune system diseases. Normal serum levels in 5–12 ng/ml and cerebrospinal fluid is generally lower than 2 ng/ml. When brain tissue is injured in varying degrees due to trauma, tumor, stroke, and poisoning, the cell membrane losses its integrity, resulting in the rapid release of NSE in damaged neurons to cerebrospinal fluid and peripheral blood [23]. It is even considered that it can be employed to predict the degree of CNS injury, and not only for various types of brain trauma, but also for diseases that cause pathological changes in brain structure and metabolism, the level of serum NSE is still related to the degree of brain injury [24]. Clinical or permissible detection of serum markers to assist in the diagnosis of SLE is not only beneficial to indirectly evaluate the severity of SLE disease, but also can be employed to evaluate the prognosis of SLE, which can provide laboratory references for early intervention [25]. When neurons are edema, degeneration, and necrosis, NSE can be released into cerebrospinal fluid and blood. Many scholars have confirmed that the level of serum NSE is significantly increased in cerebrovascular disease, neonatal hypoxic-ischemic encephalopathy, epilepsy, cardiogenic hypoxic-ischemic brain injury, brain trauma, and other diseases [26]. Among the data of our study, one patient with SLE had no neuropsychiatric symptoms and signs on admission, but his serum NSE increased significantly, and she had a major seizure 2 days later. The results of the study also indicated that the level of serum NSE in patients with SLE was significantly higher compared to the control group, and the level of NSE was closely related to the SLEDAI score, suggesting that there was a certain degree of CNS damage in patients with SLE. SLEDAI score is an important index for clinical evaluation of lupus activity. The higher the score, the stronger the lupus activity. Lupus encephalopathy often occurs in the active stage, which is a clinical sign of critical condition. However, because of its hidden performance and no specific and sensitive diagnostic methods, it is often ignored in clinical work, so it cannot be treated timely. The change of serum NSE level can early and specifically reflect the pathological changes of CNS during SLE, so it may be employed as a sensitive index to judge the existence of lupus encephalopathy in the early stage so as to provide a theoretical basis for the prevention and treatment of lupus encephalopathy [26]. At present, the detailed mechanism of the increase of serum NSE in SLE is not well understood, but it is believed to be mainly related to the following: (1) a variety of autoantibodies (antinuclear antibodies and anti-brain cell antibodies) combine with corresponding antigens to form immune complexes with the complements, which deposit on the blood vessel wall to cause cerebral vasculitis; (2) some small emboli form heart valve vegetations; (3) anticardiolipin antibodies directly act on the phospholipid components of vascular endothelial cells and platelets, leading to small thrombosis, causing microinfarction, hemorrhage, edema and brain tissue softening, and other factors, which lead to CNS lesions in patients with SLE, resulting in the increase of serum NSE levels, but the course and prognosis of NSE and SLE need to be further studied [27].

In the past decades, some serum markers have been discovered for the diagnosis and efficacy judgment of SLE, such as S100 protein. S100 protein is an acidic calcium-binding protein discovered in the mid-1960s, which originally found by Moore BW in bovine brain tissue [28]. S100 protein is considered to be a calcium-sensitive protein, which regulates biological activity through calcium binding [29]. In addition, some S100 members have been indicated to bind to zinc and/or copper, suggesting that their biological activity may be regulated by these metals in some cases. In the S100 protein family, S100B protein is the most important and most active member. In the late 1970s, F. Michetti et al. first detected S100B protein extracellular [29]. They found that the level of S100B was significantly increased in the cerebrospinal fluid of patients with multiple sclerosis in the acute phase, while the level of S100B protein was found to be lower in the stable phase of the disease, suggesting that this protein is not limited to nerve tissue [30,31]. Since then, its distribution has been found in specific cell types of nonneural tissue [32]. Some scholars have found that the levels of S100B mRNA in cerebral cortex and adipose tissue are very similar [33]. In addition to astrocytes and oligodendrocytes derived from the nervous system, a small amount of S100B protein exists in melanocytes, Langerhans cells, chondrocytes, adipocytes, adrenal medulla cells, and skeletal muscle satellite cells. In the CNS, a variety of cytokines are involved in the regulation of S100B expression, mainly interleukin-1 [34]. Both in vivo and in vitro experiments confirmed that IL-1 significantly stimulated the expression of S100B protein, which was more than threefold. By promoting the secretion of glial fibrillary acidic protein, S100B protein participates in promoting the division, proliferation, and activation of astrocytes, thus forming a positive feedback effect on astrocytes [34]. Other cytokines such as interleukin-6 and tumor necrosis factor-*α* can also promote the expression of S100B protein.

S100B protein, like most S100 members, is a calcium receptor protein that is activated at the moment of binding to calcium ions [35]. S100B is involved in the regulation of energy metabolism, transcription, protein phosphorylation, cell proliferation, survival, differentiation, and movement and calcium homeostasis [34,35]. The biological function of extracellular S100B plays different roles through the change of concentration. Physiological dose of S100B can mediate intercellular communication and intracellular signal transduction and promote the growth of glial cells and the development and maintenance of central nervous system. An animal experimental study found that S100B enhanced nerve regeneration and plasticity by promoting hippocampal synaptogenesis and synaptic formation after traumatic brain injury [36]. And S100B can significantly promote the activation of microglia after craniocerebral injury, and the duration is as long as 5 weeks. In the case of increased concentration, S100B may have neurotoxic effect by inducing cell apoptosis, causing astrocytes to release proinflammatory cytokines and nitric oxide, and then leading to oxidative stress. Therefore, the increased concentration not only reflects tissue damage, but also may aggravate tissue damage. In the experimental model of traumatic brain injury, the expression of S100B protein and mRNA increased in the injured tissue. In the same model, inhibition of the protein can reduce behavioral and pathological changes [32]. Moreover, intracerebroventricular injection of S100B has also been reported to induce dentate neurogenesis and improve cognitive function in the model of experimental traumatic brain injury (lateral fluid shock) [31]. It was found that the level of S100B in patients with SLE was significantly higher compared to the control group, suggesting that the increase of S100B protein was related [35]. The results of our current study indicated that the level of serum S100B protein in patients with SLE was not only significantly higher compared to healthy controls, but also higher compared to SLE stable group and active group, but there was no significant difference between SLE stable group and active group.

In summary, serum S100B and NSE are very sensitive indicators for judging the damage of central nervous system. However, due to the small number of cases in this study, there are as many as 19 kinds of NPSLE classification, so the relationship between serum S100B protein, NSE levels, and various NPSLE and their exact application value in diagnosing the disease and judging the prognosis needs to be confirmed by expanding the number of cases.

## Figures and Tables

**Table 1:** Comparison of general data of two groups of patients (*n*/%).

Group	C group (*n* = 60)	R group (*n* = 60)			*χ* ^*2*^	*P*
		Mild activity group	Moderate activity group	Severe activity group		
Gender (male/female)	33/27	12/8	10/10	9/11	0.133	＞0.05
Age (years)						
＜45	24 (40.00)	8 (40.00)	8 (40.00)	4 (20.00)	5.169	＞0.05
45–59	25 (41.67)	7 (35.00)	4 (20.00)	7 (35.00)		
≥60	11 (18.33)	5 (25.00)	8 (40.00)	9 (45.00)		
Marital status						
Unmarried	19 (31.67)	6 (30.00)	3 (15.00)	6 (30.00)	0.656	＞0.05
Married/divorced	41 (68.33)	14 (70.00)	17 (85.00)	14 (70.00)		
Degree of education						
Primary school and below	21 (35.00)	4 (20.00)	6 (30.00)	7 (35.00)	4.374	＞0.05
Middle school	20 (33.33)	11 (55.00)	10 (50.00)	10 (50.00)		
College or above	19 (31.67)	5 (25.00)	4 (20.00)	3 (15.00)		

**Table 2:** Comparison of serum S100B protein and NSE levels between the two groups [$\bar{x}$ ±*s*].

Group	*N*	Serum S100B protein （*μ*g/L）	Serum NSE levels（*μ*g/L）
C group	60	0.07 ± 0.01	8.28 ± 2.12
R group	60	0.22 ± 0.04	15.83 ± 3.12
*t*		28.180	15.503
*p*		<0.01	<0.01

**Table 3:** Comparison of serum S100B protein and NSE levels in patients with SLE with different activity [$\bar{x}$ ±*s*].

Group	*N*	Serum S100B protein （*μ*g/L）	Serum NSE levels (*μ*g/L）
Mild activity group	20	0.13 ± 0.02	10.28 ± 2.23
Moderate activity group	20	0.22 ± 0.04	15.83 ± 3.42
Heavy activity group	20	0.35 ± 0.07	17.94 ± 2.56
*F*		106.376	40.447
*p*		<0.01	<0.01

## Data Availability

The data used to support this study are unavailable due to patiets privacy.
